# Trajectories of part-time work and sickness absence among shift working healthcare employees

**DOI:** 10.1093/eurpub/ckac130.092

**Published:** 2022-10-25

**Authors:** A Ropponen, J Ervasti, M Härmä

**Affiliations:** 1 Finnish Institute of Occupational Health, Työterveyslaitos, Finland; 2 Division of Insurance Medicine, Karolinska Institutet, Stockholm, Sweden

## Abstract

**Background:**

Healthcare systems are facing major challenges due to population ageing, increased need for care, and economic challenges combined with staff shortage. The existing need for longer work careers combined with increasing turnover rates in healthcare highlights the need to understand working hours in association with work capacity and sustainable work careers. We aimed to investigate the concurrent changes in part-time work and sickness absence (SA) among healthcare employees without any SA spell >14 days at baseline.

**Methods:**

Annual working hour and SA data from 23 hospital districts and cities in Finland for 2008-2019 (172 922 employees with at least one work shift in any year). The sample was restricted to 20274 employees with ≤31 work shifts/year in 3 consequent years during the follow-up and without any SA spell >14 days at baseline in 2008. Part-time work/year (yes/no), SA months/year, and nightwork/year (% of nightwork of all shifts) as time varying covariate were used in the group-based trajectory models examining the concurrent changes. Models for age groups (in 2008 and categorized into < 25 years of age, ≥25 and <40 years, ≥40 and <55 years, and >55 years) will be considered later.

**Results:**

A five-trajectory solution identified groups for “stable full-time work without SA” (56.8%), “increasing part-time work and stable very low SA” (13.5%), “slight increase both in part-time work and SA” (16.5%), “steep increase in part-time work and reversed low U-shape in SA months” (5.0%), and “stable part-time work and low SA” (8.2%) across 2009-2019.

**Conclusions:**

These initial findings indicate that while most employees work full-time without SA, those who transfer from working full-time to part-time during follow-up from 2009 to 2019 seem to have low SA. Thus, part-time work may promote work capacity, and accompanied by part-time work disability benefits, offer a tool for employers to support sustainable working life and to keep older employees at work.

**Key messages:**

- Part-time work may provide possibility to maintain work participation.
- An important public health message is to provide possibilities for part-time work accompanied with partial work disability benefits to support sustainable working life.

